# Incidence of Dissociation With Low-Dose Pre-hospital Ketamine in Geriatric Patients With Trauma-Related Pain

**DOI:** 10.7759/cureus.27698

**Published:** 2022-08-05

**Authors:** Melanie M Randall, Jennifer Raae-Nielsen, Mia Choi, William S Dukes, Timothy Nesper, Michael K Mesisca

**Affiliations:** 1 Emergency Medicine, Riverside University Health System Medical Center, Moreno Valley, USA; 2 Emergency Medicine/Pharmacy, Riverside University Health System Medical Center, Moreno Valley, USA; 3 Emergency Medicine, Loma Linda University Medical Center, Loma Linda, USA

**Keywords:** pre-hospital, pain management, trauma, ketamine, geriatric

## Abstract

Introduction

Sub-dissociative ketamine has been used increasingly for the treatment of acute pain in a wide variety of settings. While there are studies evaluating its use in the geriatric population, no studies have described its safety in the pre-hospital geriatric trauma patient. The objective of this study was to define the incidence of dissociation with low-dose pre-hospital ketamine in geriatric trauma patients.

Methods

Using our county emergency medical services database, we identified all trauma patients greater or equal to 65 years of age who received pre-hospital ketamine for pain after the implementation of a low-dose ketamine protocol. We retrospectively reviewed pre-hospital and emergency department records for demographics, traumatic injuries, Glasgow Coma Score, emergency department length of stay and disposition, and hospital length of stay. This group was compared to a similar population of trauma patients, transported prior to the ketamine protocol. The comparison group was chosen by matching the ketamine group to more than double the number of non-ketamine patients. Records were obtained from two separate trauma centers. Our primary outcome was documentation of “ketamine-related dissociation” by either the pre-hospital, emergency department or trauma provider. Secondary outcomes included emergency department length of stay and intensive care unit admission.

Results

Seventy-nine patients received ketamine with records available for analysis. One hundred ninety-three non-ketamine patients were compared to this group. There were nosignificant differences between the two groups in regards to age, weight, gender, or mechanism of injury. The injury severity score was higher in the ketamine group. Nine patients (11%) had documented dissociation after ketamine, with six of these patients back to baseline mentation by arrival to the emergency department. The emergency department length of stay was shorter in the ketamine group. The rate of intensive care unit admission was the same between both groups.

Conclusions

Pre-hospital sub-dissociative ketamine in geriatric trauma patients is associated with brief episodes of dissociation in a small minority of patients.

## Introduction

Ketamine is an N-methyl-D-aspartate receptor antagonist (NMDA) with a wide variety of effects. While most commonly used for sedation, ketamine has been increasingly used and studied in the treatment of acute pain, exacerbations of chronic pain, trauma, behavioral emergencies, and depression [1-9]. In sub-dissociative doses, it has the potential to treat pain without altering mentation.

Regarding the safety of low-dose ketamine, previous studies report a wide range in the incidence of side effects that include, most commonly, vomiting, need for intubation, dissociation or over-sedation, agitation, hallucinations, and emergence reactions [6,8,10-18]. While some studies have reported zero serious adverse events, other studies have reported a moderate number of patients with one or more of the above side effects.

Increasingly, ketamine has been used in the pre-hospital setting [1,3,4,6-11,13,18-21]. Indications include rapid sequence intubation by air medical crews, traumatic and nontraumatic pain management, and treatment for behavioral emergencies. Given the physiologic property of maintaining blood pressure, ketamine may be ideal for trauma patients. However, the risk of ketamine-induced dissociation and subsequent difficulty in obtaining an accurate trauma examination are concerns in this group.

While there are studies that have evaluated ketamine in the geriatric population and demonstrated both safety and efficacy [22-25], no studies to date have specifically studied the safety of pre-hospital ketamine in geriatric trauma patients. Our hypothesis for this study is that the incidence of dissociation after pre-hospital low-dose ketamine in geriatric trauma patients is low.

## Materials and methods

We performed a retrospective cohort study comparing geriatric patients who received pre-hospital sub-dissociative intravenous (IV) ketamine for traumatic pain to a similar group of trauma patients who did not receive ketamine. On June 1, 2018, the emergency medical services (EMS) system in our county began a protocol for pre-hospital sub-dissociative IV ketamine for pain. The protocol used doses of 0.3 milligrams (mg) per kilogram (kg), with a maximum of 30 mg. This was diluted in 50-100 milliliters of normal saline and administered over five minutes. Doses were allowed to be repeated once. We identified patients who had received ketamine from the EMS agency database. 

We included all patients greater or equal to 65 years of age who presented to the emergency department (ED) of two separate trauma centers for injury over the 18-month period after implementation of the ketamine protocol and received low-dose ketamine. Per EMS protocol, all patients that received sub-dissociative ketamine had documentation of administration, dosage, and vital signs before and after ketamine was given. Patients were excluded if they received ketamine in the emergency department, or if they received ketamine for rapid sequence intubation by critical care transport. The remaining patients were then compared to a similar population of trauma patients transported prior to the protocol. The two groups were matched by age and gender. We compared the ketamine group to approximately double the number of non-ketamine patients.

We gathered the following information from both EMS and ED documentation: demographic data, trauma mechanism, injuries, Glasgow Coma Score (GCS), vital signs, Injury Severity Score (ISS), ED length of stay, disposition, intensive care unit (ICU) admission, and hospital length of stay.

The primary outcome was documentation of “ketamine-related dissociation” by either the EMS, ED, or trauma provider. Secondary outcomes included ED length of stay and incidence of ICU admission.

Data were analyzed with STATA 16 (Stata Corp., College Station, TX, USA). The Wilcoxon rank sum test was used for non-parametric data, and p<0.05 was considered significant. Descriptive statistics were also used. This study was approved by the institutional review board of both institutions.

## Results

Seventy-nine patients received ketamine and met the inclusion criteria for analysis. One hundred ninety-three non-ketamine patients were chosen for comparison. Between the two groups, there were no significant differences between age, weight, or gender (Table 1). The Injury Severity Score (ISS) was higher in the ketamine group (Table 1). The mechanisms of injury distribution were similar between the ketamine and non-ketamine groups. 

The ED length of stay was shorter in the ketamine group at a median of 385 minutes (interquartile range, IQR: 265-548 minutes) versus in the non-ketamine group at a median of 528 minutes (IQR: 327-805 minutes), p=0.0018. Rates of ED disposition to either ICU, non-ICU admission, discharge, or transfer were the same between the two groups (Table 1). Two patients in the non-ketamine group died; no patients in the ketamine group died.

**Table 1:** Patient characteristics. MOI: mechanism of injury, MVC: motor vehicle crash, MCC: motorcycle crash, ED: emergency department, LOS: length of stay, ICU: intensive care unit. *Data presented as median (interquartile range).

	Ketamine: number=79	Non-Ketamine: number=193	P value
Age in years*	75 (68-83)	78 (70-84)	0.1609
Weight in kilograms*	71 (60-90.7)	70.5 (57.7-81.1)	0.7707
Male gender	22 (28%)	64 (33%)	0.4395
MOI			
Fall	56 (71%)	142 (73%)	0.6456
MVC/MCC	15 (20%)	36 (19%)	
Other	7 (9%)	15 (8%)	
Injury severity score*	9 (5-10)	5 (1-10)	0.0072
ED LOS in minutes*	385 (265-548)	528 (327.5-805)	0.0018
Disposition			
Discharge	15 (19%)	29 (15%)	0.6279
Non-ICU	43 (54.5%)	125 (65%)	
ICU	13 (16.5%)	32 (16.5%)	
Transfer	8 (10%)	5 (2.5%)	
Death	0	2 (1%)	

The median dose of pre-hospital ketamine given was 27 mg (interquartile range (IQR): 21-30). Among the ketamine group, 20 of 79 (25%) had repeat doses, and nine of 79 (11%) ketamine patients had reported dissociation from the provider (Table 2). All nine patients that had documented dissociation received only one dose of pre-hospital ketamine. Six of the nine patients were back to their baseline mentation by arrival to the ED. There were no documented deviations from the EMS protocol.

**Table 2:** Patients with recorded dissociation after ketamine. MOI: mechanism of injury, Mg: milligrams, GCS: Glasgow Coma Score, ED: emergency department, LOS: length of stay, Min: minutes, TP: transverse process, fx: fracture. *Records state the patient was confused immediately after ketamine, although the next documented GCS was 15.

Age in years, gender	MOI	Injury	Ketamine dose (mg)	GCS before ketamine	GCS after ketamine	Back to baseline mentation prior to ED arrival?	ED LOS (min)	Disposition
74-year-old female	Fall	Spine TP fxs, hip contusion	18	15	14	No	523	Admit for two days
67-year-old female	Dog bite	Multiple lacerations	26	15	14	Yes	114	Admit for eight days
65-year-old female	Fall	Tibia/fibula fx	30	15	15*	Yes	481	Admit ICU on one day and six days admit
92-year-old female	Fall	Head injury	21	15	14	Yes	978	Admit for three days
65-year-old female	Fall	Hip pain	15	15	14	Yes	512	Discharge
81-year-old male	Fall	Shoulder dislocation	22.5	15	14	Yes	502	Discharge
83-year-old female	Fall	Femur fx	13.5	15	15*	No	238	Admit for four days
74-year-old male	Fall	Hip fx	30	15	12	Yes	548	Transfer
87-year-old female	Fall	Multiple pubic rami fx	30	14	Not documented	Not documented	700	Admit for 10 days

## Discussion

This is the first study to describe the incidence of dissociation of geriatric trauma patients receiving sub-dissociative pre-hospital ketamine for trauma-related pain. The geriatric population is increasing significantly in the United States and is projected to grow from what was 12% of the population in the year 2000 to greater than 20% in 2050 [26]. An expected increase in geriatric trauma numbers has been observed and documented [27], and trauma care for the elderly will continue to grow.

The treatment of pain in the elderly can be challenging due to changes in physical functioning, drug metabolism, and underlying medical co-morbidities. Studies have shown that providers consistently undertreat pain in this group [28]. Ketamine is at the forefront of analgesia in a growing number of clinical situations. However, there are few studies investigating its use and adverse effects in elderly patients.

In our study, the use of ketamine was not associated with an increased ED length of stay or ICU admission. The ED length of stay was surprisingly less for the ketamine group. The cause of this difference is uncertain. While there was no difference between the groups with regard to age, gender, and mechanism of injury, we did use two groups that were temporally distinct: one before the ketamine EMS protocol began, and one after.

The main limitation to our study is that being retrospective, we had to use the surrogate of a provider’s report of dissociation instead of an objective test. The findings in this study should prompt further prospective studies. Other limitations to the study include that there was a difference in the ISS found between the two groups and that the study may not have been adequately powered to find other differences. 

At 11%, the incidence of dissociation was low and brief, as evidenced by the fact that most patients recovered prior to arriving at the ED. Undertreating pain in the elderly itself has been associated with delirium, independent of opiate use [29,30]. We would argue that the benefits of analgesia outweigh the low reported incidence of dissociation after the administration of low-dose ketamine for trauma-related pain.

## Conclusions

Pre-hospital sub-dissociative ketamine in geriatric trauma patients is associated with a small incidence of dissociation. These episodes are in a minority of patients and usually resolve prior to arrival to the ED. The use of pre-hospital ketamine does not appear to be associated with a prolonged ED stay. Low-dosage ketamine appears to be a safe adjunct in this group.
